# Statewide evaluation of COVID-19 vaccine hesitancy in Rhode Island

**DOI:** 10.1371/journal.pone.0268587

**Published:** 2022-06-01

**Authors:** Brooke G. Rogers, Jun Tao, Alexi Almonte, Emily Toma, Katherine Nagel, Robert Fain, Siena C. Napoleon, Michaela A. Maynard, Matthew Murphy, Indra Neil Sarkar, Philip A. Chan

**Affiliations:** 1 Department of Medicine, Warren Alpert Medical School of Brown University, Providence, Rhode Island, United States of America; 2 Department of Psychiatry and Human Behavior, Warren Alpert Medical School of Brown University, Providence, Rhode Island, United States of America; 3 The Miriam Hospital, Providence, Rhode Island, United States of America; 4 Rhode Island Quality Institute, Providence, Rhode Island, United States of America; 5 Center for Biomedical Informatics, Brown University, Providence, Rhode Island, United States of America; 6 Department of Behavioral and Social Sciences, Brown School of Public Health, Providence, Rhode Island, United States of America; The University of Jordan, JORDAN

## Abstract

**Background:**

Vaccines are effective in preventing Coronavirus Disease 2019 (COVID-19). Vaccine hesitancy defined as delay of acceptance or refusal of the vaccine is a major barrier to effective implementation.

**Methods:**

Participants were recruited statewide through an English and Spanish social media marketing campaign conducted by a local news station during a one-month period as vaccines were becoming available in Rhode Island (from December 21, 2020 to January 22, 2021). Participants completed an online survey about COVID-19 vaccines and vaccine hesitancy with constructs and items adopted from the Health Belief Model.

**Results:**

A total of 2,007 individuals completed the survey. Eight percent (n = 161) reported vaccine hesitancy. The sample had a median age of 58 years (interquartile range [IQR]: 45, 67), were majority female (78%), White (96%), Non-Hispanic (94%), employed (58%), and reported an annual individual income of $50,000 (59%). COVID-19 vaccine hesitancy was associated with attitudes and behaviors related to COVID-19. A one unit increase in concern about COVID-19 was associated with a 69% (Adjusted Odds Ratio: 0.31, 95% CI: 0.26–0.37) decrease in vaccine hesitancy. A one-level increase in the likelihood of getting influenza vaccine was associated with a 55% (AOR: 0.45 95% CI: 0.41–0.50) decrease in vaccine hesitancy.

**Conclusions:**

COVID-19 vaccine hesitancy was relatively low in a state-wide survey in Rhode Island. Future research is needed to better understand and tailor messaging related to vaccine hesitancy.

## Introduction

Severe acute respiratory syndrome coronavirus 2 (SARS-CoV-2) is the cause of Coronavirus Disease 2019 (COVID-19) [1]. Restrictions and mitigation efforts have been broadly implemented internationally and within the United States (U.S.) to help reduce transmission [2, 3]. Vaccines against COVID-19 have demonstrated significant efficacy in disease prevention and are likely to yield great benefit if implemented effectively at the population level [4, 5]. Effective implementation of COVID-19 vaccines relies on supply and logistics associated with delivery and administration, as well as on behavioral factors, including willingness of the population to receive the vaccine [6].

A potential challenge to effective COVID-19 vaccine implementation is vaccine hesitancy, which continues to be a major public health concern with vaccine implementation at a population level [7]. For the purposes of this paper, we have adopted the Strategic Advisory Group of Experts on Immunization (SAGE) ‘s definition of vaccine hesitancy as defined in MacDonald et al., 2015, which defines vaccine hesitancy in terms of context, time, and place, and recognizes the nuance of vaccine hesitancy and acceptance along a continuum ranging from “accepting all” to “refusing all” vaccines [8]. Applying the SAGE Working Group’s definition of vaccine hesitancy encourages us to explore the correlates of vaccine hesitancy related to the COVID-19 vaccine that are situated within the sociocultural context of this current pandemic [8].

Vaccination during prior emergent public health crises (e.g. polio vaccine) have relied on increasing acceptability for vaccination. The Health Belief Model, originally developed in the 1950s by a group of social psychologists in the U.S. Public Health Service to explain why individuals do not participate in disease prevention programs, offers one explanation of how individuals make decisions about engagement in a specific health-promoting behavior. The Health Belief Model suggests that individuals are influenced by: the benefits and drawbacks of receiving the vaccine, the threats of acquiring the illness, their own self-efficacy (or belief they can engage in the behavior), and external or environmental factors (including cues to action reminding them to engage in the behavior) [9, 10]. Because the COVID-19 vaccine is emerging in direct response to an acute public health crisis and individuals are being exposed to several cues to action, including through mainstream media and social media, the Health Belief Model is an appropriate theory from which to position understanding willingness and hesitancy for vaccination. Additionally, while the perceived benefits are avoiding illness or more serious complications of the illness, there are still several perceived barriers. The most notable barriers are concerns about the novelty or “newness” of the vaccine and the lack of longitudinal data [11]. Thus, we use the Health Belief Model to frame our understanding of the current climate around COVID-19 vaccination as well as the findings within this study.

Globally, trends in willingness to accept a COVID-19 vaccine have been varied [12]. A study conducted online across 19 countries with a panel of over 13,000 respondents found that in countries where there was a strong trust in central government (e.g. China, South Korea, Singapore), there were very low rates of vaccine hesitancy [13]. Across all countries, those with lower education and income levels had higher vaccine hesitancy, suggesting the need for information that is easy to understand and approaches to dissemination of information from locally trusted sources to address community-level concerns. A systematic review of COVID-19 vaccine hesitancy in the U.S. conducted by Yasmin and colleagues (14) between 2020 and 2021 found that vaccine hesitancy was highest among Black/African Americans and pregnant or breastfeeding women and lowest among the male sex. They also found regional differences within the U.S. (though most were based on one or two small studies which raises questions in their validity and reliability) and subpopulation differences based on occupation (e.g. healthcare, military), social roles (e.g. pregnant women, prison/jail inmates), health status (e.g. patients with diseases, tobacco/marijuana users) and affiliations (e.g. religious groups). They also found changes over time, with hesitancy rising significantly between 2020 and 2021, suggesting wider acceptance was growing as more individuals were vaccinated for COVID-19 [14].

Findings from a prior study of a nationally representative sample of U.S. adults (N = 991) in a cross-sectional survey found that 58% intended to be vaccinated, 32% were unsure, and 11% did not intend to be vaccinated. Vaccine hesitancy was associated with a younger age, Black race, lower socioeconomic status (SES), and not having received the flu vaccine [15]. Reasons for not being vaccinated included concerns about side-effects, safety, and efficacy, the need for additional information, anti-vaccine attitudes and beliefs such as misconceptions, and lack of trust. Importantly, African American/Black, Hispanic/Latino, and other communities of color have been significantly more likely to display vaccine hesitancy due to historical mistrust and maltreatment [16, 17].

The goal of the current study was to examine vaccine hesitancy in Rhode Island at the start of vaccine availability in the state (December 2020-January 2021). This study aimed to capture vaccine hesitancy and associated attitudes during the early phases of vaccine implementation and to identify specific beliefs, demographic characteristics, and other factors that may be associated with vaccine hesitancy.

## Methods

### Participants and procedures

Participants were recruited through a statewide media campaign launched with Nexstar Communications, which is a U.S.-based publicly traded media company. Locally, Nexstar has a news station, WPRI, which is a leading local news source and website in both Rhode Island and Southeastern Massachusetts with coverage that reaches over 600,000 households. Contrary to larger, national news stations, we are not aware of any political affiliations or biases (conservative, moderate, or liberal) associated with this local news station, which also made it an ideal choice for this partnership. Our recruitment strategy included having WPRI place study-specific, IRB approved banners on a local news station’s webpage, co-branded media posts on a local news station’s Facebook page, and direct-to-consumer Facebook advertisements. All direct-to-consumer advertisements placed on Facebook were presented in both English and Spanish. Advertisements ran for one month, starting December 21, 2020 and ending January 22, 2021.

Advertisements in English and Spanish were equivalent in language. The English language advertisement read: “*Thoughts about the COVID-19 vaccine*?*”* The Spanish advertisement read, “*¿Qué piensa usted sobre la vacuna contra el COVID-19*?*”* The banner ad on the media pages for the local news station stated *"Thoughts about the COVID-19 vaccine*? *Take our research survey*.*”* Co-branded advertisements, which explained that researchers were interested in community members’ thoughts about the vaccine and then displayed the same advertisement, were also posted by the local news station. Advertisements were posted continuously and received comments, shares, and interactions beyond their posting date. Data on the impact and reach of the campaign was collected by WPRI and Nexstar Digital. Per their records, as of January 21, 2021, the Facebook post on December 22, 2020 reached a cumulative 63,013 unique people, had 928 post reactions (likes, loves, comments, etc.), 8,229 post clicks (photo clicks, link clicks, etc.), and 650 direct links to the survey page. The Facebook post on January 7, 2021 reached a cumulative 48,499 unique people, had 595 post reactions (likes, loves, comments, etc.), 1,541 post clicks (photo clicks, link clicks, etc.), and 496 direct links to the survey page. In total, the Facebook post reached over 110,000 Rhode Islanders. The banner ads had 110,003 impressions and 38 clicks to the survey page for a click-through rate of 0.03%. Due to the low click-through rate on the banner ads, remaining funding was shifted to Spanish language Facebook advertisements mid-campaign (first week of January).

The Rhode Island Hospital Institutional Review Board approved a waiver of written informed consent. Participants did provide informed consent, but name and signature were not required as the study posed no more than minimal risk and providing anonymity allowed participants to share their thoughts freely. Prior to seeing the survey questions, all participants were presented with information about the study, decided if they wanted to participate, and selected “Yes/No” from the electronic survey. A portion of individuals selected “No” and then continued to answer survey questions. These answers were dropped from the analyses. All study procedures were approved by the Rhode Island Hospital Institutional Review Board.

### Data collection

Once individuals clicked the advertisement link, they were redirected to a survey and selected to take the survey in their language of preference (English or Spanish). Survey data were collected using REDCap (Research Electronic Data Capture), which is a secure, HIPAA-compliant, web-based application designed to support data capture for research studies [18]. Data were collected and stored within a secure server of the REDCap application in a project accessible to members of the research team. All questions were asked of all participants and most required a selection to advance. However, individuals were able to select an option of “decline to answer” for all questions. The following data were collected to better understand factors associated with vaccine hesitancy:

#### Demographic information

Participants were asked to provide the following demographic information: age, sex assigned at birth, current gender identity, sexual orientation, race, ethnicity, and personal income. Additionally, participants were asked for their employment status and if they had experienced any period of lost employment due to COVID-19. Finally, those who indicated they had received a COVID-19 PCR test at any time were asked if the results were positive or negative.

#### COVID-19 vaccine hesitancy/willingness

The main outcome variable was COVID-19 vaccine hesitancy/willingness. Participants were asked, “If there was a vaccine that could prevent COVID-19 in the future, how likely would you be to get the vaccine?” Answer choices were on a Likert-type scale and included the following options: not at all likely –0, not likely– 1, somewhat likely– 2, likely– 3, or very likely– 4. For regression analyses, the Likert scale answer choices were collapsed into a dichotomous outcome coded such that vaccine hesitant/unwilling = 1 (which included not at all likely and not likely) and not vaccine hesitant/willing = 0 (which included somewhat likely, likely, and very likely answer choices). This was done to facilitate ease of interpretation and clinically meaningful results.

#### Flu vaccine hesitancy/willingness

Participants were asked, “What is the probability that you receive the flu vaccine this year?” Answer choices were on a Likert-type scale and included the following options: not at all likely –0, not likely– 1, somewhat likely– 2, likely– 3, or very likely– 4. There was also the option: “I already received the flu vaccine this year.”

#### Concerns about COVID-19 vaccination

Participants were asked, “What are some reason(s) you may not want the vaccine?” This question provided a set of options in a checklist and was marked as “check all that apply.”

#### Concerns about COVID-19 infection

Participants were asked, “What are some reasons you are concerned about being infected with SARS-CoV-2?” This question provided a set of options in a checklist and was marked as “check all that apply.”

### Data analysis

Bivariate analysis examined relationships between demographic variables and willingness to receive the COVID-19 vaccine (Yes vs. No). Chi-square and Kruskal-Wallis tests were used to test the distribution for categorical and continuous variables by willingness to receive the COVID-19 vaccine, respectively. Logistic regression models were used to assess associations between variables of interest and willingness to receive the COVID-19 vaccine. Odds ratios and corresponding confidence intervals were calculated, and adjusted odds ratios and corresponding confidence intervals accounted for covariates of age, gender, race, and ethnicity. Confounding variables were determined by a *priori* and directed acyclic graphs. The analyses were conducted using Stata 16.0. (StataCorp LLC, College Station, TX).

## Results

### Demographic characteristics

A total of 3,048 people responded to the survey. We excluded 71 individuals who clicked on the survey but declined participation, and 963 individuals who did not complete the survey. The remainder of the participants numbered 2,014. However, we further excluded seven participants who did not respond to the survey item that asked about their willingness to take the COVID-19 vaccine. The final cohort of participants for our analytic sample was 2,007. The sample had a median age of 58 years old (interquartile range [IQR]: 45, 67), were majority female (78%), White (96%), non-Hispanic (94%), employed (58%), and reported an annual individual income of $50,000 (59%). Eleven percent of the sample reported losing their job during COVID-19 and returning to work and 8% percent had lost their jobs and were looking for work at the time of survey. Of the total sample, 58% had a negative PCR test and 8.1% had a positive PCR test. However, only 66% (n = 1,326) of the sample had been tested for COVID-19 via of a PCR test; and, of these, 12% had a positive test. Approximately 72% of the total sample had already had their annual influenza vaccine at the time of completing the survey. There was a fairly disperse distribution of concern about getting COVID-19 with most respondents indicating at least some concern or greater. Additional demographic and behavioral characteristics are reported in Table 1.

**Table 1:** Sample characteristics by COVID-19 vaccine willingness.

Variables		COVID-19 vaccine willingness					
	Total (N = 2,007)		Yes		No		*P* value
			N	%	N	%	
Age (median, IQR)	58 (45, 67)		59 (46, 67)		47.5 (34, 58)		<0.001
**Sex**							0.238
Male	437	21.8%	396	21.5%	41	25.5%	
Female	1,569	78.2%	1,449	78.5%	120	74.5%	
**Race**							0.47
White	1,894	94.4%	1,750	96.1%	144	94.1%	
Black/African American	11	0.5%	10	0.5%	1	0.7%	
Asian/Pacific Islander/Other	69	3.4%	61	3.3%	8	5.2%	
**Ethnicity**							0.896
No, not Hispanic/Latino	1,858	92.6%	1,713	93.8%	145	93.5%	
Yes, Hispanic/Latino	123	6.1%	113	6.2%	10	6.5%	
**Income (U.S. dollars, USD)**							<0.001
<30K	451	22.5%	398	21.6%	53	32.7%	
30K-50K	365	18.2%	327	17.7%	38	23.5%	
50K-100K	758	37.8%	714	38.7%	44	27.2%	
>100K	433	21.6%	406	22.0%	27	16.7%	
**Employment**							<0.001
Full/part-time	1,174	58.5%	1,057	57.3%	117	72.2%	
Unemployed/disabled/other	253	12.6%	224	12.1%	29	17.9%	
Retired	553	27.6%	539	29.2%	14	8.6%	
Student	27	1.3%	25	1.4%	2	1.2%	
**Loss of Employment**							0.001
No	1,613	80.4%	1,499	81.2%	114	70.4%	
Yes, but return to work	241	12.0%	207	11.2%	34	21.0%	
Yes, looking for jobs	153	7.6%	139	7.5%	14	8.6%	
**COVID PCR test results (n = 1,326)**							0.082
Negative	1,163	57.9%	1,091	88.1%	72	81.8%	
Positive	163	8.1%	147	11.9%	16	18.2%	
**Likelihood of Flu vaccination**							<0.001
Not at all	216	10.8%	102	5.5%	114	70.4%	
Not likely	35	1.7%	28	1.5%	7	4.3%	
Somewhat likely	38	1.9%	34	1.8%	4	2.5%	
Likely	57	2.8%	56	3.0%	1	0.6%	
Very likely	217	10.8%	216	11.7%	1	0.6%	
Already received	1,441	71.8%	1,406	76.3%	35	21.6%	
**Concerned about COVID-19**							<0.001
Not at all	181	9.0%	104	5.6%	77	47.5%	
A little	254	12.7%	213	11.5%	41	25.3%	
Somewhat concerned	346	17.2%	326	17.7%	20	12.3%	
Concerned	550	27.4%	540	29.3%	10	6.2%	
Very much concerned	676	33.7%	662	35.9%	14	8.6%	

**Key**: N = number in the sample, IQR = Interquartile Range (25%-75%), P-values obtained either by Chi-Square or Kruskal-Wallis tests of significance.

### Vaccine hesitancy versus willingness for COVID-19 vaccine

Vaccine hesitancy and willingness were interpreted as inverse descriptions along the same continuum of attitudes towards vaccines. We coded data to interpret vaccine attitudes in terms of hesitancy. A small portion of our sample (N = 161; 8%) reported some vaccine hesitancy (operationalized as “Not at all likely” or “Not likely” response to willingness to get the vaccine). In our sample, vaccine hesitancy did not differ by gender, race, and ethnicity. However, we had limited diversity in the sample and were therefore not able to draw any comparisons between subgroups. Those of younger age and having an individual annual income less than $50,000 USD were more likely to report vaccine hesitancy when compared to their older and wealthier counterparts. Individuals who reported their employment status as “retired” had low vaccine hesitancy, and 97.5% were willing to be vaccinated. Individuals who received the flu vaccine showed extremely low rates of vaccine hesitancy with 97.6% reporting willingness to receive the COVID-19 vaccine.

### Reasons against COVID-19 vaccination and concern about COVID-19

There were statistically significant associations between vaccine hesitancy and beliefs about COVID-19 and vaccination safety. Participants who were concerned about COVID-19 were more likely to be willing to be vaccinated; 97.9% of those who were “very concerned” and 98.2% of those who were “concerned” reported willingness to get vaccinated (see Table 1).

At the bivariate level, there were statistically significant associations between various concerns about COVID-19 illness and willingness to be vaccinated. Individuals who endorsed the following were more likely to express vaccine willingness: being concerned about things that cannot be controlled, being concerned about feeling ill and uncomfortable, not wanting to be in the hospital, not wanting to get others sick, and being afraid of dying (Table 2).

**Table 2:** Reasons for COVID-19 concern.

Reasons for COVID-19 Concern	COVID-19 vaccine willingness						
	Total (N = 2,007)		Yes		No		*P* value
		%	N	%	N	%	
In general, I am someone who gets concerned about things I cannot control							<0.001
No	1,409	70.2%	1,268	68.73%	141	87.04%	
Yes	598	29.8%	577	31.27%	21	12.96%	
I will feel very ill and uncomfortable							<0.001
No	1,226	61.1%	1,087	58.92%	139	85.80%	
Yes	781	38.9%	758	41.08%	23	14.20%	
I will not be able to breathe well and I hate feeling out of breath							<0.001
No	1,187	59.1%	1,054	57.13%	133	82.10%	
Yes	820	40.9%	791	42.87%	29	17.90%	
I do not want to be in the hospital							<0.001
No	858	42.8%	744	40.33%	114	70.37%	
Yes	1,149	57.2%	1,101	59.67%	48	29.63%	
I do not want to get others sick							<0.001
No	402	20.0%	315	17.07%	87	53.70%	
Yes	1,605	80.0%	1,530	82.93%	75	46.30%	
I am afraid of dying							<0.001
No	1,196	59.6%	1,056	57.24%	140	86.42%	
Yes	811	40.4%	789	42.76%	22	13.58%	
I am not concerned at all							<0.001
No	1,881	93.7%	1,783	96.64%	98	60.49%	
Yes	126	6.3%	62	3.36%	64	39.51%	

**Key**: N = number in the sample, IQR = Interquartile Range (25%-75%), P-values obtained either by Chi-Square or Kruskal-Wallis tests of significance.

At the bivariate level, there were significant associations between endorsement of reasons not to obtain the vaccine and vaccine hesitancy. Specifically, individuals who endorsed the following were less likely to express vaccine willingness: not being worried about COVID-19, concern that vaccines were questionably safe, concern that there was not enough information about COVID-19 vaccines, concerns that vaccines have negative side effects, belief that vaccines are part of a suspicious government program, and other (Table 3).

**Table 3:** Reasons for COVID-19 vaccination hesitancy.

Reasons for COVID-19 Vaccine Hesitancy	COVID-19 vaccine willingness						
	Total (N = 2,007)		Yes		No		*P* value
			N	%	N	%	
I am not worried about getting COVID-19							<0.001
No	1,889	94.1%	1,777	96.31%	112	69.14%	
Yes	118	5.9%	68	3.69%	50	30.86%	
Vaccines are still questionably safe							<0.001
No	1,778	88.6%	1,694	91.82%	84	51.85%	
Yes	229	11.4%	151	8.18%	48	48.15%	
Vaccines are expensive							0.607
No	1,912	95.3%	1,759	95.34%	153	94.44%	
Yes	95	4.7%	86	4.66%	9	5.56%	
Not enough information about COVID vaccine							<0.001
No	1,552	77.3%	1,514	82.06%	38	23.46%	
Yes	455	22.7%	331	17.94%	124	76.54%	
Already received too many vaccines							<0.001
No	1,988	99.1%	1,835	99.46%	153	94.44%	
Yes	19	0.9%	10	54.00%	9	5.56%	
Vaccines can have negative side effects							<0.001
No	1,625	81.0%	1,546	83.79%	79	48.77%	
Yes	382	19.0%	299	16.21%	83	51.23%	
Vaccines have needles, which can be painful and/or scary							0.299
No	1,947	97.0%	1,792	97.13%	155	95.68%	
Yes	60	3.0%	53	2.87%	7	4.32%	
Vaccines are part of a government program, and I am suspicious of the government							<0.001
No	1,908	95.1%	1,811	98.16%	97	59.88%	
Yes	99	4.9%	34	1.84%	65	40.12%	

**Key**: N = number in the sample, IQR = Interquartile Range (25%-75%), P-values obtained either by Chi-Square or Kruskal-Wallis tests of significance.

### Multivariate logistic regression models

The focus of this study was to explore factors associated with vaccine hesitancy. As such, individuals who reported no vaccine hesitancy were used as the reference group. Higher income was associated with lower vaccine hesitancy when adjusted for age, gender, race, and ethnicity. With regards to age, each one-year increase in age was associated with a 4% (crude odds ratio [cOR]: 0.96, 95% confidence interval [CI]: 0.95–0.97) decrease in vaccine hesitancy. Females were 20% (cOR: 0.80, 95% CI: 0.55–1.16) less likely than males to report vaccine hesitancy. In the adjusted model, retired people were 60% (adjusted odds ratio [aOR]: 0.40, 95% CI: 0.20–0.78) less likely to express vaccine hesitancy when we further adjusted for age, gender, race, and ethnicity. A one-level increase in the likelihood of getting the flu vaccine was associated with a 55% (aOR: 0.45 95% CI: 0.41–0.50) decrease in vaccine hesitancy. A one-level increase in level of the concern of COVID-19 was associated with a 69% (aOR: 0.31, 95% CI: 0.26–0.37) decrease in vaccine hesitancy (Table 4).

**Table 4:** Factors predicting COVID-19 vaccine hesitancy.

Variables	COVID-19 Vaccine Hesitancy	
	Crude odds ratio (95% confidence interval)	Adjusted odds ratio (95% confidence interval)
**Age**	0.96 (0.95, 0.97)	.
**Sex**		
Male	Ref^1^	Ref
Female	0.80 (0.55, 1.16)	.
**Race**		
White	Ref	Ref
Black/African American	1.22 (0.15, 9.56)	.
Asian/Pacific Islander/Other	1.59 (0.75, 3.40)	.
Ethnicity		
No	Ref	Ref
Yes	1.05 (0.54, 2.04)	.
**Income** *		
<30K	Ref	Ref
30K-50K	.	0.89 (0.54, 1.46)
50K-100K	.	0.43 (0.26, 0.69)
>100K	.	0.50 (0.29, 0.86)
**Employment** *		
Full/part-time	Ref	Ref
Unemployed/disabled/other	.	0.90 (0.55, 1.49)
Retired	.	0.40 (0.20, 0.78)
Student	.	0.27 (0.06, 1.22)
**Loss Employment during COVID** *		
No	Ref	Ref
Yes, but return to work	.	1.36 (0.86, 2.17)
Yes, looking for jobs	.	0.99 (0.53, 1.85)
**COVID PCR test results** *		
Negative	Ref	Ref
Positive	.	1.28 (0.65, 2.53)
**Likelihood to get flu vaccine** *	.	0.45 (0.41, 0.50)
**Concerns about infection** *	.	0.31 (0.26, 0.37)

Key:

* Adjusted for age, gender, race, and ethnicity; The focus of this study was to explore factors associated with vaccine hesitancy. As such, individuals who reported no vaccine hesitancy were used as the reference group.

## Discussion

This study is among the first to evaluate vaccine hesitancy during the early phases of COVID-19 vaccine implementation in the U.S. These results demonstrate a low degree of vaccine hesitancy in a statewide survey associated with various demographic and behavioral characteristics. Older individuals, retirees, and females had lower levels of vaccine hesitancy. Individuals who were highly concerned about COVID-19 and/or had received the influenza vaccine also reported very low levels of vaccine hesitancy.

The health belief model has been used to understand preventive health behaviors [9] and has been applied to influenza [19] human papilloma virus (HPV) [20], and COVID-19 vaccination [21]. The model posits that individuals weigh the perceived benefits and perceived threats of a health behavior prior to engaging in the behavior. Perceived threats consist of perceived severity, or how problematic the given health outcome would be, and perceived susceptibility, or how likely it is to have that given health outcome. Cues to action from the environment also contribute to the decision to engage in that health behavior.

Our results suggest that those who had a high level of concern for acquiring COVID-19 (e.g. high perceived severity/perceived susceptibility) were least likely to express vaccine hesitancy. Additionally, those who had already had the flu vaccine had significantly lower vaccine hesitancy (perhaps suggesting that for them, the perceived benefits of vaccination, generally, outweigh the perceived threats). With regards to age and COVID-19 vaccine hesitancy specifically, our results suggest less perceived severity and susceptibility of COVID-19 (and greater vaccine hesitancy) among younger individuals compared to older individuals, which accurately reflects hospitalization, morbidity, and mortality rates [22]. However, younger individuals are more likely to be involved in activities outside the home including school, work, roommate living situations, and social activities, thereby representing a population that could unknowingly, and perhaps asymptomatically, transmit the disease to more vulnerable members of society [22, 23].

Vaccines are critical for reducing the spread of infectious diseases [24], and studies have indicated that vaccine hesitancy affects a heterogenous group of people [15, 25]. Anti-vaccine messaging is pervasive on websites, online forums, video and content creation platforms, and social media [26–29]. Viewing these sources for a short time (i.e. 5–10 minutes) can be associated with an increased perception of vaccination risks and decreased behavioral intentions to obtain vaccinations [30, 31]. Anti-vaccine arguments are often grounded in anecdotal experiences, which are amplified through media. Among these arguments are the debunked myths that vaccines cause autism or other neurological disorders [32] or that vaccines contain harmful ingredients such as thiomersal [17, 25]. Nevertheless, we acknowledge that the COVID-19 vaccination efforts have the additional burden of having to prove that these vaccines which have been developed and delivered to the public on an abbreviated timeline are efficacious. The lack of longitudinal data on COVID-19 vaccines presents the additional challenge to the medical and public health sectors with having to assure the public of vaccine safety.

Vaccine hesitancy can be affected by contextual, group, individual, and vaccine specific factors [8]. Significant medical mistrust, especially among communities of color in the U.S., may also play a role in vaccine hesitancy more broadly and as it relates to the COVID-19 vaccine [16, 33]. Historical reasons for increased vaccine hesitancy in minority populations have included higher rates of medical mistrust, lower access to preventative health, out-of-pocket costs, and other social- and structural-level barriers to care [16, 17, 34]. Complicating matters, in the past there have been adverse incidents related to vaccines [7, 27]. Future studies and public health initiatives need to focus on addressing common misbeliefs about vaccines and working with communities to engage those who have been historically marginalized and better develop interventions to address vaccine hesitancy.

At the public health level, several initiatives have been conducted to address vaccine hesitancy. The U.S. Department of Health and Human Services [35] Centers for Disease Control and Prevention (CDC) [17], CDC Foundation [36], and state and local departments of public health, including The Rhode Island Department of Health [37], have developed media and social media campaigns to improve education and awareness about the COVID-19 vaccines. Additionally, theory-based approaches implemented in other parts of the world may be useful in approaching vaccine hesitancy for COVID-19 vaccines in the U.S. For example, the Tailoring Immunizations Programme (TIP), a product of the SAGE Working Group on Vaccine Hesitancy, has been successfully used to plan and implement vaccines in marginalized and vaccine hesitant groups including Somali immigrant communities in Sweden and Orthodox Jewish communities in the United Kingdom [38].

The strengths of this study must be interpreted in light of its limitations. Data collected for this study were from a statewide convenience sample utilizing a combination of Facebook and digital media advertising through a local news station. Thus, although efforts were made to cast a wide net, the sample we collected does not represent the fullest range of individuals or identities in the state. We generated our own measures of items around vaccine hesitancy and COVID-19 concern that were not previously tested or validated psychometrically. However, we weighed the potential negative consequences of this approach with the need to obtain information in a timely manner due to the immediate public health needs. Data for this study were also collected at the very beginning of vaccine availability and may differ from what we might expect to find if the study were conducted at a later time. Additionally, the data we collected have the same limitations as all cross-sectional data including the inability to examine temporal relationships and infer causality from results. A major limitation of the current study is the low number of Non-White participants. We attempted to engage Spanish-speaking populations with the use of Spanish-language advertising and were successful in reaching additional participants; however, Spanish speakers were still underrepresented, as were Hispanic/Latino populations overall when compared to state demographics. Low participation in studies is an additional challenge in understanding reasons for vaccine hesitancy in these groups. Without engagement, we are underpowered to draw any meaningful conclusions about minority subgroups. We are planning to organize focus groups with Hispanic/Latino participants to better understand their willingness to receive COVID-19 vaccination. This will enable detailed analyses to characterize any differences between the general population survey and specific populations.

In conclusion, our data represent an initial snapshot of attitudes towards vaccination in the state of Rhode Island. Overwhelmingly, vaccine hesitancy was limited, and individuals were interested in vaccination and extremely willing to be vaccinated. Future research should explore whether individual attitudes towards vaccination directly predicted whether that individual chose to be vaccinated. Given the low rates of participation among racially and ethnically diverse individuals in our study we cannot draw any conclusions specific to minority communities. Future research should be focused on understanding vaccine hesitancy within a variety of communities to help promote solutions that increase health equity.

## Supporting information

S1 Data (PDF)

S2 Data (CSV)

S3 Data (CSV)
