# Supplementary material for: Statewide evaluation of COVID-19 vaccine hesitancy in Rhode Island
Source: PLoS One. 2022 Jun 1;17(6):e0268587. doi: 10.1371/journal.pone.0268587 (PMC9159551; doi:10.1371/journal.pone.0268587)
Supplement: S1 Data — (PDF) [file pone.0268587.s001.pdf]

Codebook

Data Dictionary Codebook

02/01/2022 6:56am

Collapse all instruments

	#	Variable / Field Name	Field Label *Field Note*	Field Attributes (Field Type, Validation, Choices, Calculations, etc.)
Instrument: **COVID-19 Testing Survey** (covid19_testing_survey) Enabled as survey Collapse				
	1	record_id	Record ID	text

	2	elements_consent	Understanding COVID-19 Testing and Vaccination in Rhode Island<br><br>We would like to ask you to take part in a research study called, Understanding COVID-19 Testing and Vaccination in Rhode Island.<br><br>The goals of this study are to determine:<br>1) how individuals in Rhode Island feel about different COVID-19 testing options<br>2) the contributing factors that influence that decision, and<br>3) willingness to be vaccinated against COVID-19 if a vaccine were eventually developed<br><br>You are being asked to participate in this study because you reside in Rhode Island. Your responses will help us plan for COVID-19 testing in Rhode Island.<br><br>We expect to conduct at least 1,000 online surveys.<br><br>If you choose to participate in the study, you will be asked to complete an online survey. The survey is confidential. The survey asks you about your demographic information, how you feel about COVID-19 testing options, contributing factors that influence that decision, and willingness to be vaccinated against COVID-19 if a vaccine were eventually developed. The survey should take no longer than 10 minutes to complete.<br><br>You will not be reimbursed for your participation in this research study.<br><br>There are no costs to you for participating in this study.<br><br>Your part in this research study consists of completing a survey. This study does not require you to have procedures or treatments. Therefore, being in this study does not involve any physical risks to you. However, there is a slight risk regarding the confidentiality of your participation in this study, if information about you becomes known to persons outside this study. The researchers are required to keep your study information confidential, however, so the risk of breach of confidentiality is very low.<br><br>Study data will be shared only in a summary format. This ensures that none of the information you provide will be linked back to you.<br><br>Your participation in this study may not benefit you personally. The indirect benefit is that the results of this study will help to inform the development of strategies for promoting COVID-19 testing. Therefore, the results of this study have the potential to inform public health strategies to contain COVID-19 including preferences for making testing (if it becomes available) vaccination accessible.<br><br>Participation in this study is completely voluntary. You are not required to enroll or participate. If you decide to participate, you can always change your mind and quit at any time.<br><br>If you have any questions about the research study, please feel free to contact Brooke G. Rogers, Ph.D., M.P.H., at 401-793-7077.<br><br>If you have any questions about your rights as a research subject please feel free to call our Research Protections Office Director, Janice Muratori, at (401) 444-6246.	descriptive

	3	agree_participate	If you decide to participate in the study, you are agreeing to the components of the informed consent process. You can print a copy of this document for your records.	radio, Required<br>0 No, I do not want to participate<br>1 Yes, I agree to participate<br><br>Custom alignment: LV<br>Stop actions on 0
	4	covid_age	Section Header: *Survey Progress: 0% The purpose of this survey is to help plan for COVID-19 testing and vaccination in Rhode Island. This survey is anonymous and will not be linked to you personally. Please answer each question. Demographics We are interested in your characteristics to better understand who COVID-19 is affecting in Rhode Island.*<br>How old are you?	text (integer), Required
	5	covid_sex_birth	What was your sex assigned at birth?	radio, Required<br>1 Male<br>2 Female<br>3 Other:
	6	covid_sex_birth_other<br><br>Show the field ONLY if:<br>[covid_sex_birth] = '3'	Other (please specify):	text
	7	covid_gender	What is your current gender identity?	radio, Required<br>1 Man<br>2 Woman<br>3 Transgender man<br>4 Transgender woman<br>5 Other:<br>6 Decline to answer
	8	covid_gender_other<br><br>Show the field ONLY if:<br>[covid_gender] = '5'	Other (please specify):	text
	9	covid_sexual_orientation	What is your sexual orientation?	radio, Required<br>1 Heterosexual<br>2 Bisexual<br>3 Lesbian/Gay<br>4 Pansexual<br>5 Asexual<br>6 Other:<br>7 Decline to answer
	10	covid_sexual_orientation_other<br><br>Show the field ONLY if:<br>[covid_sexual_orientation] = '6'	Other (please specify):	text
	11	covid_race	What is your race?	checkbox, Required<br>1 covid_race___1 White<br>2 covid_race___2 Black or African American<br>3 covid_race___3 American Indian or Alaska Native<br>4 covid_race___4 Asian<br>5 covid_race___5 Native Hawaiian or other Pacific Islander<br>6 covid_race___6 Other:<br>7 covid_race___7 Decline to answer
	12	covid_race_other_2<br><br>Show the field ONLY if:<br>[covid_race(6)] = '1'	Other (please specify):	text

	13	covid_ethnicity	Are you Hispanic/Latino?	radio, Required<br>0 No<br>1 Yes<br>2 Decline to answer
	14	covid_zip	What ZIP Code do you live in?	text (zipcode), Required
	15	covid_household	How many people including you live in your household?	text (integer), Required
	16	covid_employment_status	What is your current employment status?	radio, Required<br>1 Full-time (30 or more hours per week)<br>2 Part-time (fewer than 30 hours per week)<br>3 Unemployed<br>4 Retired<br>5 Disabled, not able to work<br>6 Student<br>7 Other:
	17	covid_employment_status_other<br>Show the field ONLY if:<br>[covid_employment_status] = '7'	Other (please specify):	text
	18	covid_loss_employment	Was there a period of time you were not working due to COVID-19?<br>*Since March 1, 2020*	radio, Required<br>1 No<br>2 Yes, but I am now back at the job I had previously<br>3 Yes, but I am now back at a different job<br>4 Yes, I lost my previous job and now looking for work<br>5 Yes, I was laid off my previous job, but plan to start back work at the same job in the future
	19	covid_loss_employment_length<br>Show the field ONLY if:<br>[covid_loss_employment] = '2' or [covid_loss_employment] = '3' or [covid_loss_employment] = '4' or [covid_loss_employment] = '5'	If yes, how long have you or had you been out of work due to COVID-19?<br>Number of weeks:<br>*By out of work we mean not working. This does not include if you have been working remotely from home and getting paid.*	text (integer)
	20	covid_income	This past year, what was your total personal income before taxes?	radio, Required<br>1 < $10,000<br>2 $10,001-$20,000<br>3 $20,001-$30,000<br>4 $30,001-$40,000<br>5 $40,001-$50,000<br>6 $50,001-$60,000<br>7 $60,001-$70,000<br>8 $70,001-$80,000<br>9 $80,001-$90,000<br>10 $90,001-$100,000<br>11 $100,001-$110,000<br>12 $110,001-$120,000<br>13 $120,001-$130,000<br>14 $130,001-$140,000<br>15 $140,001-$150,000<br>16 $150,001+

	21	covid_job_setting	What type of setting do/did you work in (select one)?	radio, Required<br>1 Healthcare<br>2 Dental<br>3 Childcare<br>4 Congregate living (Nursing home, Assisted living, Group home)<br>5 Faith-based organization<br>6 Gym<br>7 Hair<br>8 Personal services (Nail / Massage / Tattoo / Facialist / Other)<br>9 Physical therapy<br>10 Restaurant or Bar<br>11 Grocery store<br>12 Retail<br>13 Manufacturing<br>14 Education<br>15 Government (Inspectors, DMV, others)<br>16 Public transportation<br>17 First responder (Fire/EMS/Police)<br>18 Corrections<br>19 Other:
	22	covid_job_setting_other<br>Show the field ONLY if:<br>[covid_job_setting] = '19'	Other (please specify):	text
	23	covid_work_zip<br>Show the field ONLY if:<br>[covid_employment_status] = '1' or [covid_employment_status] = '2' or [covid_employment_status] = '7'	What ZIP Code do you work in?	text (zipcode)
	24	covid_transportation	What modes of transportation do you use during a typical week (check all that apply)?	checkbox, Required<br>1 covid_transportation___1 Personal vehicle<br>2 covid_transportation___2 Carpool<br>3 covid_transportation___3 Rideshare<br>4 covid_transportation___4 Bus<br>5 covid_transportation___5 Train<br>6 covid_transportation___6 Bicycle<br>7 covid_transportation___7 Walking<br>8 covid_transportation___8 Other:
	25	covid_transportation_other<br>Show the field ONLY if:<br>[covid_transportation(8)] = '1'	Other (please specify):	text
	26	covid_country_travel	Section Header: *Survey Progress: 0% Contact with COVID-19. We would like to know if you suspect you have had or had contact with someone who has been infected with SARS-CoV-2, the virus that causes COVID-19.*<br>Have you traveled out of the country since January 1, 2020?	yesno, Required<br>1 Yes<br>0 No
	27	covid_country_travel_yes<br>Show the field ONLY if:<br>[covid_country_travel] = '1'	If yes, location and date(s):	notes
	28	covid_state_travel	Have you traveled outside of the state since January 1, 2020?	yesno, Required<br>1 Yes<br>0 No

	29	covid_state_travel_yes<br><br>Show the field ONLY if:<br>[covid_state_travel] = '1'	If yes, location and date(s):	notes
	30	covid_contact	Have you had close contact with a known COVID-19 positive case?<br>*Close contact is defined as within six feet for at least 10-15 minutes. (If you are a healthcare worker, this means being in close contact without proper personal protective equipment.)*	yesno, Required<br>1 Yes<br>0 No
	31	covid_contact_yes<br><br>Show the field ONLY if:<br>[covid_contact] = '1'	If yes, relationship and date(s):	notes
	32	covid_quarantine	Have you been instructed to be in quarantine or isolation due to COVID-19 by the Rhode Island Department of Health or a medical provider?	yesno, Required<br>1 Yes<br>0 No
	33	covid_quarantine_yes<br><br>Show the field ONLY if:<br>[covid_quarantine] = '1'	If yes, date(s):	notes
	34	covid_household_positive	Has anyone you live with (in your household) tested positive for COVID-19?	yesno, Required<br>1 Yes<br>0 No
	35	covid_household_positive_yes<br><br>Show the field ONLY if:<br>[covid_household_positive] = '1'	If yes, relationship and date(s):	notes
	36	covid_exposure	Do you think you may have been exposed to COVID-19?	yesno, Required<br>1 Yes<br>0 No
	37	covid_exposure_yes<br><br>Show the field ONLY if:<br>[covid_exposure] = '1'	If yes, date(s):	notes
	38	covid_symptoms	Have you had any symptoms of COVID-19 since January 1, 2020 including today?	yesno, Required<br>1 Yes<br>0 No
	39	covid_symptoms_yes<br><br>Show the field ONLY if:<br>[covid_symptoms] = '1'	Which symptoms (check all that apply):	checkbox<br>1 covid_symptoms_yes___1 Fever<br>2 covid_symptoms_yes___2 Chills<br>3 covid_symptoms_yes___3 Cough<br>4 covid_symptoms_yes___4 Trouble breathing<br>5 covid_symptoms_yes___5 Runny/stuffy nose<br>6 covid_symptoms_yes___6 Abdominal pain<br>7 covid_symptoms_yes___7 Headache<br>8 covid_symptoms_yes___8 Sore throat<br>9 covid_symptoms_yes___9 Diarrhea<br>10 covid_symptoms_yes___10 Nausea/vomiting<br>11 covid_symptoms_yes___11 Fatigue<br>12 covid_symptoms_yes___12 Muscle pain<br>13 covid_symptoms_yes___13 Loss taste/smell<br>14 covid_symptoms_yes___14 Other symptoms:
	40	covid_symptoms_yes_other<br><br>Show the field ONLY if:<br>[covid_symptoms_yes(14)] = '1'	Other symptoms (please list):	text

	41	covid_likelihood	How likely do you think it is that you've had COVID-19 in the past?	radio, Required<br>1 Not at all likely<br>2 Not likely<br>3 Somewhat likely<br>4 Likely<br>5 Very likely
	42	covid_offered_pcr	Section Header: *Survey Progress: 0% COVID-19 Testing We would like to know what you think about testing for SARS-CoV-2, the virus that causes COVID-19.*<br>Have you previously had a swab test of the nose for SARS-CoV-2?(i.e. either a nasal or nasopharyngeal swab)?	yesno, Required<br>1 Yes<br>0 No
	43	if_yes_how_many_times<br>Show the field ONLY if:<br>[covid_offered_pcr] = '1'	IF YES, How many times?	text
	44	where_were_you_tested<br>Show the field ONLY if:<br>[covid_offered_pcr] = '1'	Where were you tested?	radio<br>1 Primary care provider or community clinic<br>2 Urgent care center<br>3 Emergency Department<br>4 State-site through portal.ri.gov<br>5 My place of work<br>6 Other
	45	where_were_you_tested_other<br>Show the field ONLY if:<br>[where_were_you_tested] = '6'	Please specify:	text
	46	covid_pcr_results<br>Show the field ONLY if:<br>[covid_offered_pcr] = '1'	Have you every had a positive test?	radio<br>0 No<br>1 Yes
	47	covid_pcr_likelihood	If you were offered a swab test of your nose for SARS-CoV-2 today, how likely would you be to get one?	radio, Required<br>1 Not at all likely<br>2 Not likely<br>3 Somewhat likely<br>4 Likely<br>5 Very likely
	48	covid_pcr_reasons_yes	What are some reasons you WOULD be willing to get tested? (check all that apply)	checkbox, Required<br>1 covid_pcr_reasons_yes___1 I would want to know for my own health<br>2 covid_pcr_reasons_yes___2 I would want to know so I don't transmit to others<br>3 covid_pcr_reasons_yes___3 It is free<br>4 covid_pcr_reasons_yes___4 It does not take much effort<br>5 covid_pcr_reasons_yes___5 It could inform public health measures<br>6 covid_pcr_reasons_yes___6 It is required to travel<br>7 covid_pcr_reasons_yes___7 It is required for work<br>8 covid_pcr_reasons_yes___8 Other:
	49	covid_pcr_reasons_yes_other<br>Show the field ONLY if:<br>[covid_pcr_reasons_yes(8)] = '1'	Other (please specify):	text

	50	covid_pcr_reasons_no	What are some reasons you WOULD NOT be willing to get tested (check all that apply)?	checkbox, Required<br>1 covid_pcr_reasons_no___1 I am concerned the test will be uncomfortable<br>2 covid_pcr_reasons_no___2 I am concerned about the costs of the test<br>3 covid_pcr_reasons_no___3 I do not want to know<br>4 covid_pcr_reasons_no___4 I do not think I have been exposed<br>5 covid_pcr_reasons_no___5 I do not and have not had symptoms<br>6 covid_pcr_reasons_no___6 I do not trust the accuracy of the tests<br>7 covid_pcr_reasons_no___7 I already had COVID-19<br>8 covid_pcr_reasons_no___8 I am not concerned about COVID-19<br>9 covid_pcr_reasons_no___9 I do not have access to transportation<br>10 covid_pcr_reasons_no___10 I do not have access to childcare<br>11 covid_pcr_reasons_no___11 I have to work and I am unable to get off of work<br>12 covid_pcr_reasons_no___12 I am concerned about the possibility of becoming infected when I go for a test<br>13 covid_pcr_reasons_no___13 If I tested positive, people might judge me<br>14 covid_pcr_reasons_no___14 If I tested positive, I would have to isolate/self-quarantine<br>15 covid_pcr_reasons_no___15 I was recently tested<br>16 covid_pcr_reasons_no___16 Other:
	51	covid_pcr_reasons_no_other<br>Show the field ONLY if:<br>[covid_pcr_reasons_no(16)] = '1'	Other (please specify):	text
	52	covid_offered_ab	Have you previously had a blood test to see if you have the antibodies to SARS-CoV-2?	yesno, Required<br>1 Yes<br>0 No
	53	covid_ab_results<br>Show the field ONLY if:<br>[covid_offered_ab] = '1'	What were the results of this test?	radio<br>1 Negative<br>2 Positive<br>3 Inconclusive<br>4 Don't know

<table>
<tr><td></td><td>54</td><td>covid_ab_likelihood</td><td>If you were offered a blood test to see if you have antibodies to SARS-CoV-2 today, how likely would you be to get one?</td><td>radio, Required<table><tr><td>1</td><td>Not at all likely</td></tr><tr><td>2</td><td>Not likely</td></tr><tr><td>3</td><td>Somewhat likely</td></tr><tr><td>4</td><td>Likely</td></tr><tr><td>5</td><td>Very likely</td></tr></table></td></tr>
<tr><td></td><td>55</td><td>covid_ab_reasons_yes</td><td>What are some reasons you WOULD be willing to get tested? (check all that apply)</td><td>checkbox, Required<table><tr><td>1</td><td>covid_ab_reasons_yes___1</td><td>I would want to know for my own health</td></tr><tr><td>2</td><td>covid_ab_reasons_yes___2</td><td>I would want to know so I don't transmit to others</td></tr><tr><td>3</td><td>covid_ab_reasons_yes___3</td><td>It is free</td></tr><tr><td>5</td><td>covid_ab_reasons_yes___5</td><td>It does not take much effort</td></tr><tr><td>6</td><td>covid_ab_reasons_yes___6</td><td>It could inform public health measures</td></tr><tr><td>7</td><td>covid_ab_reasons_yes___7</td><td>Other:</td></tr></table></td></tr>
<tr><td></td><td>56</td><td>covid_ab_reasons_yes_other
Show the field ONLY if: [covid_ab_reasons_yes(7)] = '1'</td><td>Other (please specify):</td><td>text</td></tr>
</table>

	57	covid_ab_reasons_no	What are some reasons you WOULD NOT be willing to get tested? (check all that apply)	checkbox, Required<br>1 covid_ab_reasons_no___1 I am concerned the test will be painful<br>2 covid_ab_reasons_no___2 I am concerned about the costs of the test<br>3 covid_ab_reasons_no___3 I do not want to know<br>4 covid_ab_reasons_no___4 I do not think I have been exposed<br>5 covid_ab_reasons_no___5 I do not and have not had symptoms<br>6 covid_ab_reasons_no___6 I do not trust the accuracy of the tests<br>7 covid_ab_reasons_no___7 I already had COVID-19<br>8 covid_ab_reasons_no___8 I am not concerned about COVID-19<br>9 covid_ab_reasons_no___9 I do not have access to transportation<br>10 covid_ab_reasons_no___10 I do not have access to childcare<br>11 covid_ab_reasons_no___11 I have to work and I am unable to get off of work<br>12 covid_ab_reasons_no___12 I am concerned about the possibility of becoming infected when I go for a test<br>13 covid_ab_reasons_no___13 If I tested positive, people might judge me<br>14 covid_ab_reasons_no___14 If I tested positive, I would have to isolate/self-quarantine<br>15 covid_ab_reasons_no___15 I was already tested<br>16 covid_ab_reasons_no___16 Other:
	58	covid_ab_reasons_no_other<br>Show the field ONLY if:<br>[covid_ab_reasons_no(16)] = '1'	Other (please specify):	text
	59	covid_concern	How concerned are you about becoming infected with SARS-CoV-2? If you've previously tested positive, how concerned are you about becoming re-infected with SARS-CoV-2?	radio, Required<br>1 Not at all concerned<br>2 A little concerned<br>3 Somewhat concerned<br>4 Concerned<br>5 Very much concerned

	60	covid_concern_reasons	What are some reasons you are concerned about being infected with SARS-CoV-2? (check all that apply)	checkbox, Required<br>1 covid_concern_reasons___1 In general, I am someone who gets concerned about things I cannot control.<br>2 covid_concern_reasons___2 I will feel very ill and uncomfortable.<br>3 covid_concern_reasons___3 I will not be able to breathe well and I hate feeling out of breath.<br>4 covid_concern_reasons___4 I do not want to be in the hospital.<br>5 covid_concern_reasons___5 I do not want to get others (family, friends) sick.<br>6 covid_concern_reasons___6 I am afraid of dying.<br>7 covid_concern_reasons___7 I am not concerned at all<br>8 covid_concern_reasons___8 Other:
	61	covid_concern_reasons_other<br>Show the field ONLY if:<br>[covid_concern_reasons(8)] = '1'	Other (please specify):	text
	62	covid_concern_no	What are some reasons you are NOT concerned about becoming infected with SARS-CoV-2 ? (check all that apply)	checkbox, Required<br>1 covid_concern_no___1 In general, I try not to get concerned about things I cannot control.<br>2 covid_concern_no___2 I do not believe it is as bad as they say.<br>3 covid_concern_no___3 I am relatively healthy, so it should not affect me too much.<br>4 covid_concern_no___4 I have been very sick in the past and made it through.<br>5 covid_concern_no___5 I believe the medical teams are learning a lot and will be able to help me if I do need help.<br>6 covid_concern_no___6 Other:
	63	covid_concern_no_other<br>Show the field ONLY if:<br>[covid_concern_no(6)] = '1'	Other (please specify):	text
	64	covid_vaccine	If there was a vaccine that could prevent COVID-19 in the future, how likely would YOU be to get the vaccine?	radio<br>1 Not at all likely<br>2 Not likely<br>3 Somewhat likely<br>4 Likely<br>5 Very likely

	65	covid_vaccine_reasons	What are some reason(s) YOU may NOT want the vaccine? (check all that apply)<br>*If there are no reasons, do not mark any responses.*	checkbox<br>1 covid_vaccine_reasons___1 I am not worried about getting COVID-19<br>2 covid_vaccine_reasons___2 Vaccines are still questionably safe<br>3 covid_vaccine_reasons___3 Vaccines are expensive<br>4 covid_vaccine_reasons___4 There is not enough information about the vaccine for COVID-19<br>5 covid_vaccine_reasons___5 Already received too many vaccines<br>6 covid_vaccine_reasons___6 Vaccines can have negative side effects<br>7 covid_vaccine_reasons___7 Vaccines have needles, which can be painful and/or scary<br>8 covid_vaccine_reasons___8 Vaccines are part of a government program, and I am suspicious of the government agenda<br>9 covid_vaccine_reasons___9 Other:
	66	covid_vaccine_reasons_other<br>Show the field ONLY if: [covid_vaccine_reasons(9)] = '1'	Other (please specify):	text
	67	what_is_the_probability_th	What is the probability that you receive the flu vaccine this year?	radio<br>1 Not at all likely<br>2 A little likely<br>3 Somewhat likely<br>4 Likely<br>5 Very likely<br>6 I already received the flu vaccine this year.
	68	covid_caregiver	Section Header: *Survey Progress: 0% These next questions are about the child(ren) in your care. If there are no children in your care, no questions will appear.*<br>Are you a primary caregiver of children under the age of 18?	yesno, Required<br>1 Yes<br>0 No
	69	covid_children_pcr<br>Show the field ONLY if: [covid_caregiver] = '1'	If you were offered a swab test of the nose for SARS-CoV-2 today FOR THE CHILD(REN) IN YOUR CARE, how likely would you be to have them tested?	radio<br>1 Not at all likely<br>2 Not likely<br>3 Somewhat likely<br>4 Likely<br>5 Very likely

	70	covid_kids_ab_reasons_yes<br>Show the field ONLY if:<br>[covid_caregiver] = '1'	What are some reasons you WOULD be willing to get THE CHILD(REN) IN YOUR CARE tested? (check all that apply)	checkbox<br>1 | covid_kids_ab_reasons_yes___1 | I would want to know for their health<br>2 | covid_kids_ab_reasons_yes___2 | I would want to know so they don't transmit to others<br>3 | covid_kids_ab_reasons_yes___3 | It is free<br>4 | covid_kids_ab_reasons_yes___4 | It does not take much effort<br>5 | covid_kids_ab_reasons_yes___5 | It is required for school<br>6 | covid_kids_ab_reasons_yes___6 | Other:
	71	covid_kids_ab_reasons_yes_other<br>Show the field ONLY if:<br>[covid_kids_ab_reasons_yes(6)] = '1'	Other (please specify):	text
	72	covid_kids_ab_reasons_no<br>Show the field ONLY if:<br>[covid_caregiver] = '1'	What are some reasons you WOULD NOT be willing to get THE CHILD(REN) IN YOUR CARE tested? (check all that apply)	checkbox<br>1 | covid_kids_ab_reasons_no___1 | I am concerned the test will be painful<br>2 | covid_kids_ab_reasons_no___2 | I am concerned about the costs of the test<br>3 | covid_kids_ab_reasons_no___3 | I do not want to know<br>4 | covid_kids_ab_reasons_no___4 | I do not think they have been exposed<br>5 | covid_kids_ab_reasons_no___5 | I do not think they have had symptom[illegible]<br>6 | covid_kids_ab_reasons_no___6 | I do not trust the accuracy of the test<br>7 | covid_kids_ab_reasons_no___7 | They already had COVID-19<br>8 | covid_kids_ab_reasons_no___8 | I am not concerned about COVID-19<br>9 | covid_kids_ab_reasons_no___9 | I do not have acces[illegible] to transportation<br>15 | covid_kids_ab_reasons_no___15 | I do not have acces[illegible] to childcare<br>10 | covid_kids_ab_reasons_no___10 | I have to work, and [illegible] am unable to get o[illegible] of work<br>11 | covid_kids_ab_reasons_no___11 | I am concerned about the possibilit[illegible] of becoming infecte[illegible] when going for a te[illegible]<br>12 | covid_kids_ab_reasons_no___12 | If they tested positive, people might judge me<br>13 | covid_kids_ab_reasons_no___13 | If they tested positive, I would have to keep them isolated/quarantine[illegible]<br>14 | covid_kids_ab_reasons_no___14 | Other:
	73	covid_kids_ab_reasons_no_other<br>Show the field ONLY if:<br>[covid_kids_ab_reasons_no(14)] = '1'	Other (please specify):	text

	74	covid_vaccine_kids_likely<br><br>Show the field ONLY if:<br>[covid_caregiver] = '1'	If there was a vaccine that could prevent COVID-19 in the future, how likely would you be to get the vaccine FOR THE CHILD(REN) IN YOUR CARE?	radio<br>1 Not at all likely<br>2 A little likely<br>3 Somewhat likely<br>4 Likely<br>5 Very likely<br>6 There are no children in my care
	75	covid_vaccine_kids_not<br><br>Show the field ONLY if:<br>[covid_caregiver] = '1'	What are some reason(s) you may NOT want the vaccine FOR THE CHILD(REN) IN YOUR CARE? (check all that apply)	checkbox<br>1 covid_vaccine_kids_not___1 I am not worried about the child(ren) in my care getting COVID-19<br>2 covid_vaccine_kids_not___2 Vaccines are still questionably safe<br>3 covid_vaccine_kids_not___3 Vaccines are expensive<br>4 covid_vaccine_kids_not___4 There is not enough information about the vaccine for COVID-19<br>5 covid_vaccine_kids_not___5 Already received too many vaccines<br>6 covid_vaccine_kids_not___6 Vaccines can have negative side effects<br>7 covid_vaccine_kids_not___7 Vaccines have needles, which can be painful and/or scary<br>8 covid_vaccine_kids_not___8 Vaccines are part of a government program, and I am suspicious of the government agenda<br>9 covid_vaccine_kids_not___9 Other:
	76	covid_vaccine_kids_not_oth<br><br>Show the field ONLY if:<br>[covid_vaccine_kids_not(9)] = '1'	Other (please specify):	text
	77	covid_comments	Thank you for completing our survey. We hope that this information can be used to improve the health of Rhode Island. If you have additional other thoughts related to COVID-19, your health, and the health of your community, please share them here.	notes<br>Custom alignment: LV
	78	covid19_testing_survey_complete	Section Header: *Form Status*<br>Complete?	dropdown<br>0 Incomplete<br>1 Unverified<br>2 Complete
